# Analysis of Genetic Interaction Networks Shows That Alternatively Spliced Genes Are Highly Versatile

**DOI:** 10.1371/journal.pone.0055671

**Published:** 2013-02-07

**Authors:** David Talavera, Ritika Sheoran, Simon C. Lovell

**Affiliations:** Faculty of Life Sciences, University of Manchester, Manchester, United Kingdom; University Of Oxford, United Kingdom

## Abstract

Alternative splicing has the potential to increase the diversity of the transcriptome and proteome. Where more than one transcript arises from a gene they are often so different that they are quite unlikely to have the same function. However, it remains unclear if alternative splicing generally leads to a gene being involved in multiple biological processes or whether it alters the function within a single process. Knowing that genetic interactions occur between functionally related genes, we have used them as a proxy for functional versatility, and have analysed the sets of genes of two well-characterised model organisms: *Caenorhabditis elegans* and *Drosophila melanogaster*. Using network analyses we find that few genes are functionally homogenous (only involved in a few functionally-related biological processes). Moreover, there are differences between alternatively spliced genes and genes with a single transcript; specifically, genes with alternatively splicing are, on average, involved in more biological processes. Finally, we suggest that factors other than specific functional classes determine whether a gene is alternatively spliced.

## Introduction

Alternative splicing (AS) is common to most eukaryotes and significantly increases the diversity of the transcriptome and the proteome [1], [2], [3]. Since an alternatively spliced gene can generate multiple transcripts through differential processing of the pre-mRNA, this increase in diversity can be achieved with a relatively modest increase in the size of the genome.

The expression of specific transcripts is a complex phenomenon that involves the joint action of many proteins and ribonucleoproteins [4], [5], [6], [7], [8]. This complexity is necessary to generate the particular transcripts required for specific tissues or development stages. Some of the transcripts generated through AS seem not to be functional and are frequently removed by mechanisms such as nonsense mediated decay [9], [10]. By contrast, other transcripts perform their function as RNAs whereas others only fulfil their role when translated into proteins.

Differentially-spliced transcripts often have different molecular functions, due to the substantial changes introduced by AS [11], [12]. These different functions may result from differing interactions with other molecules. Differential interactions can be of crucial relevance in therapy [13], [14], [15], as specific peptides or transcripts can be targeted with drugs or RNAi.

Direct physical interactions between proteins are of key importance in determining molecular function [16], [17]. The increased diversity of the proteome generated through AS can produce a large variety of protein-protein interactions, which can be both time and tissue specific [18], [19], [20]. Function can also be viewed through functional interactions such as genetic interactions. Genetic interactions are detected when simultaneous gene alterations (mutation, deletion or overexpression) of two genes has a phenotypic effect that is more or less than the multiplicative effect of each alteration measured individually [21]. Although the molecular mechanism by which they occur is complex, several studies have demonstrated that genetic interactions tend to occur between functionally related genes [22], [23], [24], [25], [26], [27]; examples include genes that code for members of protein complexes or enzymes participating in the same metabolic pathway. Thus, a genetic interaction occurring between two genes is a sign that those genes are involved in similar or related biological processes.

Alternative splicing may affect networks of genetic interactions in a number of ways. First, some alternatively spliced genes might be related to several biological processes, and so would have a large number of unrelated genetic interactions. However, it is unknown whether genes with multiple transcripts are involved in more biological processes than those with a unique transcript; i.e. if all transcripts were used to finely tune the function of the alternatively-spliced gene, there would be no differences between genes with and without AS in terms of the number of biological processes genes are involved in. Secondly, genes might have many time- or tissue-specific isoforms working on the same process. Finally, if more unrelated transcripts are related to one function, this not only increases the chances of new genetic interactions, but also the probability of a functional backup effect, which could minimise previous interactions.

In this study, we employed graph theory to explore the consequences of AS upon the epistatic versatility, *i.e.,* the ability of some genes to genetically interact with other genes that are involved in many different biological processes. As not all functional relationships are obvious (e.g. parallel metabolic pathways), we used genetic interactions as a proxy for functional relatedness. We hypothesise that AS widens the range of biological processes a gene is involved in, and so increases the number of genetic interactions it has. Thus, analysing the effect of AS on the network of genetic interactions, we can infer the influence of AS on epistasis. Accordingly, we analysed the genetic interactions network of two different organisms: the nematode *Caenorhabditis elegans*, and the fruit fly *Drosophila melanogaster*. Our results show that alternatively spliced genes are more likely than genes with a single transcript to develop epistatic relationships with participants in different biological processes. Moreover, we found other differences that point towards a non-random distribution of alternatively spliced genes on the overall network of genetic interactions, suggesting that not all genes have the same probability of being alternatively spliced.

## Methods

### AS Information

We considered genes as alternatively spliced if they have the ability to generate more than one transcript, even if no functional information was available for them. Ensembl transcripts [28] were used as they are supported by experimental evidence. We used transcript information instead of peptide data, as in some cases the functional molecule could be the RNA. The use of a curated database probably leads to an underestimation of genes with AS.

### Network of Genetic Interactions

We used for the analysis all genetic interactions from two different species: *C. elegans* and *D. melanogaster* taken from BioGRID repository [29]. We built two undirected graphs where genes were the nodes of the network, and an edge was established between each pair of nodes with a reported genetic interaction, disregarding the type of genetic interaction (see Table 1). Genetic interactions are caused by a diverse range of mechanisms; e.g.: gene deletion/mutation or alteration of gene expression. Moreover, genetic interactions can produce very diverse phenotypcial outcomes; e.g.: some genetic interactions can induce lethality, whereas others will rescue a lethality phenotype. Nevertheless, this diversity does not affect the construction of the graphs since we are analysing the existence or absence of interactions. We used the genetic interactions as proxies of the functional relatedness between genes. It is known that most genetic interactions occur between genes involved in the same or parallel biological processes [22], [23], [24], [25], [26], [27], [30]. Genes acting in numerous processes are functionally related to many other genes; hence, their chances of developing genetic interactions are greater.

**Table 1:** Description of the genetic interaction networks.

	Numberof nodes	Numberof edges	% ofAS nodes
**C. elegans**	1096	2262	49.1%
**D. melanogaster**	984	5229	52.1%

### Parameters used in the Graph Analyses

#### Degree

The number of edges a node has is the degree of that node. In our case, the degree of a node corresponds to the number of genes with which the gene has epistasis. As genetic interactions mainly occur between functionally related genes [22], [23], [24], [25], [26], [27], even if they are not in the same functional module [27], [30], this parameter approximates the quantity of biological processes in which the gene is involved. Thus, the higher the degree, the more biological processes a gene may be involved.

#### Betweenness

Freeman’s betweenness of a node describes its centrality, calculating the number of shortest paths from each vertex to any other vertex that passes through that node. Nodes may have a high betweeness because they bridge different areas of the network, or because they have many interactions. In both cases, the shortest paths pass through that node because other nodes are not linked. Thus, high centrality suggests that a particular gene may be involved in many and disparate biological processes.

#### Clustering coefficient

The clustering coefficient gives information about how interconnected are a node’s neighbours. In our context, a high clustering coefficient means functional homogeneity, as most functionally related genes would also genetically interact.

#### Diameter

To calculate the diameter we calculate the shortest path between each pair of nodes. The longest of these is the diameter. If the analysed network is sparse, the diameter measure is to correspond to the diameter of the greatest subnetwork. As most of the nodes are in the greatest network component, a small diameter means that some genes must have many connections, and thus be extremely versatile.

## Results

### AS Increases the Number of Unrelated Interactions

**Figure 1:**
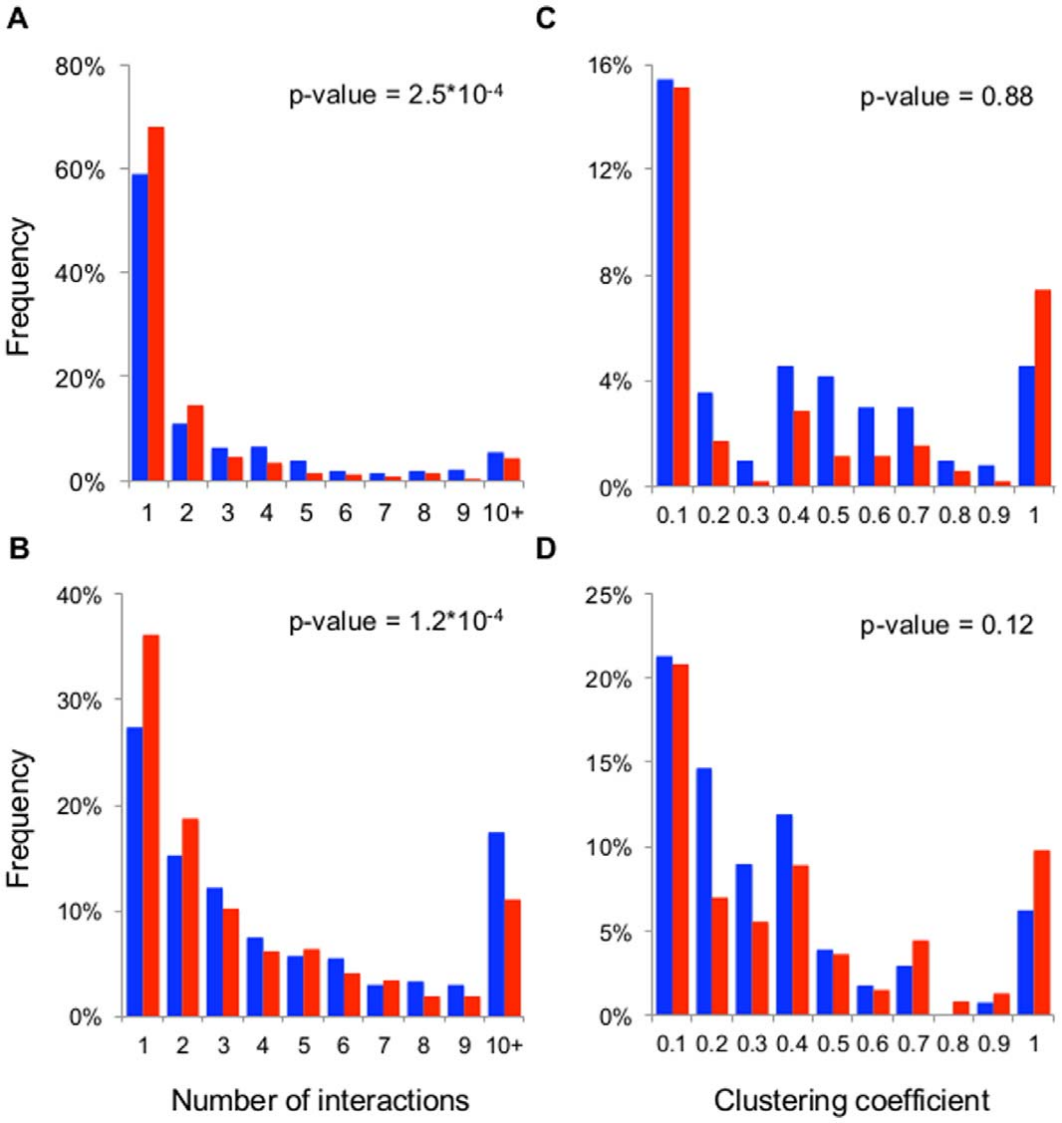
Histograms of the distributions of degree and clustering coefficient for alternatively spliced genes and genes with a single transcript. A and C, *C. elegans*. B and D, *D. melanogaster*. In blue, alternatively spliced genes. In red, genes with a unique transcript. P-values calculated using Mann-Whitney test and FDR correction for the equivalent comparisons.

Alternative splicing generates multiple transcripts from a single gene, which may differ in both function and interactions with other molecules. Thus, we hypothesise that alternatively spliced genes will have more genetic interactions on average than genes generating a unique transcript. To test this hypothesis, we calculated the degree for each node in the genetic interaction network (*i.e.*, the number of interactions). Although both classes of genes generate distributions of generally similar form (Figure 1 A,B), the Mann-Whitney test shows that the distributions are significantly different (P-value<0.05 in both cases). In both species, genes with a single transcript have fewer genetic interactions on average.

By contrast, there are no significant differences between the distributions of clustering coefficient of genes with and without AS (P-values>0.05 in both cases; Figure 1 C,D). Taken together, these results show that AS increase the number of genetic interactions, and that most of them are unrelated. Most genes with clustering coefficients equal to 0 or 1 have few interactions. The genes with more interconnected interactions are the fruit fly genes Su(var)2–10 and Dcp-1, which have 4 and 5 completely connected interactions, respectively. As expected, genes with very high degree have near-zero clustering coefficients, consistent with their role as participants in many unrelated processes.

### AS is Related to Variation in Centrality

**Figure 2:**
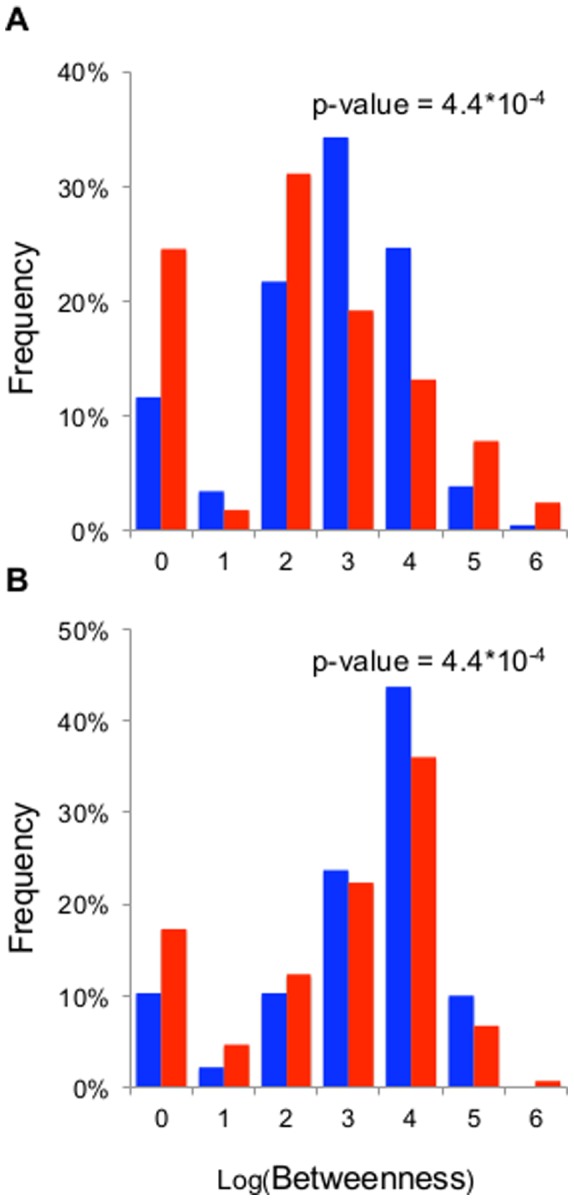
Histograms of the distribution of betweenness for alternatively spliced genes and genes with a single transcript. A, *C. elegans*. B, *D. melanogaster*. In blue, alternatively spliced genes. In red, genes with a unique transcript. P-values calculated using Mann-Whitney test and FDR correction for the equivalent comparisons.

On average, nodes with high degree have an increased probability of having high centrality, since there are many paths that pass through the node increasing the potential to link nodes that are not directly connected. In our context, genes with many genetic interactions are likely to participate in different biological processes, and may act as functional bridges. Betweenness is a centrality measure that takes into account the number of shortest paths passing through each node. The percentage of nodes having one or few shortest paths passing through them is greater for genes without AS (P-value<0.05 in both cases; Figure 2). Alternatively spliced genes have higher betweenness than genes with a unique transcript. Nonetheless, the nodes with the highest betweenness tend not to be alternatively spliced, in accordance with the existence of a few non-alternatively spliced genes having many interactions.

As expected, on average genes with large numbers of interactions have a high betweenness. However, we also found that there are some proteins that are very central despite a lower number of interactions, suggesting that they act as bridges between different functions (Figure S1). Several transcripts of the nematode’s lin-3 growth factor have 11 genetic interactions and similar betweenness as genes with more than 100 interactions. In *D. melanogaster*, the protein kinase lok (alternatively spliced) and the visual-receptor ninaE (only one transcript) are amongst the most central nodes of the network despite having just 6 and 9 interactions, respectively.

### Alternatively Spliced genes and genes with an Unique Transcript have Different Locations in the Interaction Network

These results suggest that the two sets of genes (those with AS and those with unique transcripts) make distinct contributions to the genetic interaction networks. To test this hypothesis we analysed the variation of the diameter when removing sets of nodes of particular types from the network. The network’s diameter is the shortest distance between the most separated nodes in the network. Removing nodes can affect the diameter in three different ways: 1) if the nodes are eccentric (having very low centrality), the diameter will tend to decrease because the periphery of the network will be removed; 2) if the nodes are hubs (i.e. nodes with many interactions), the diameter will increase because the shortest paths must make a detour; and, 3) if the nodes are bottlenecks, (*i.e.,* the exclusive connection between particular parts of the network), the diameter will dramatically decrease because the network will fragment. For the majority of nodes, deletion should have only a minor effect on the diameter as they are redundant in terms of finding the shortest path. For example, imagine an undirected graph containing 4 nodes forming a square. The diameter of this graph is 2 and it would remain the same after removing any one of the nodes. However, an effect of a node deletion upon the network diameter will be seen after subsequent deletions. For instance, if two nodes have a redundant role as bottlenecks, the removal of one of them, will not affect the diameter; however, the removal of the second node would split the network in two, making obvious the backup role of the first removed node.

**Figure 3:**
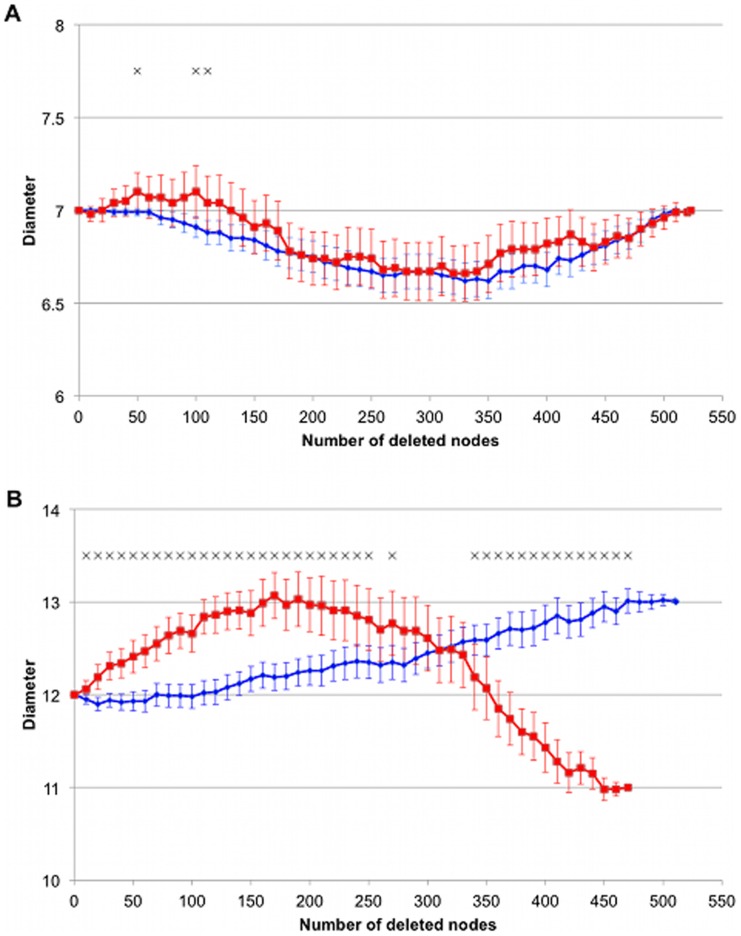
Change on the network’s diameter due to deletion of random nodes. A: *C. elegans*. B: *D. melanogaster*. Graphs show the mean and standard error of 100 experiments, deleting nodes with AS (blue) or without (red). The overall difference is tested doing a multivariate analysis of variance (MANOVA), applying Pillai-Bartlett test to the MANOVA table and adjusting the p-values using FDR correction. Only the deletion of the two sets of fruit fly transcripts generates significantly different distributions (p-value<0.05). Black crosses show which points show differences (adjusted p-value<0.05) independently of the overall result.

In both species, the initial sequential removal of alternatively spliced nodes leads to a slight decrease of the diameter. However, their series progression is different. In contrast, the removal of genes without alternative splicing shows a different picture from the very start (Figure 3). For *D. melanogaster* there are marked differences between genes alternatively spliced genes and those with a single transcript (P-value<0.05; MANOVA), whereas there are no global differences for *C. elegans* (P-value>0.05; MANOVA). Experiments deleting non-alternatively spliced genes show greater variance. This observation is in accordance with the existence of a very few non-alternatively spliced genes that are highly connected and central in the network. These nodes are likely to disproportionately affect the network despite not being frequent. By contrast, the smallest variance obtained when randomly deleting alternatively spliced nodes means that there are only small differences on the effect of removing different alternatively spliced nodes.

## Discussion

Differences between AS genes and those with a single transcript may arise directly from alternative splicing. Alternatively, these trends may be due to other confounding factors, such as AS genes having particular sets of functions. The genes with the greatest number of genetic interactions in *C. elegans* are involved in signal transduction pathways: receptors daf-2, egl-15 and let-23 are alternatively spliced, whereas lin-35, let-756, bar-1, let-60 and sem-5 have a unique transcript. lin-35, which is the *C. elegans* orthologue of the RBL2 gene, has genetic interactions with 521 different genes. This gene is involved in a wide range of biological processes, probably acting redundantly in some of the processes [31], [32], [33], [34], [35]. Thus it has a high probability of being involved in genetic interactions. In the case of fruit fly, the genes having that largest numbers of interactions are also involved in signalling pathways. However, pnr, CycE and Egfr are alternatively spliced whereas Ras85D and N are not. Signalling proteins act in many biological processes, and so are rich in genetic interactions independently of their splicing features. Thus, at least for those genes with the largest numbers of interactions, which will have a large effect on the differences in the distributions of degree (Figure 1 A,B), both classes of nodes are involved in similar functions. There is no clear link between AS and functional class; rather the differences observed are more likely to arise from the phenomenon of AS directly.

Genes with multiple interactions can be epistatically versatile, that is, they may predominantly make interactions with genes that are functionally unrelated. Alternatively, they may be functionally homogenous, characterised by their interactors also interacting with each other to form closed cliques. Figures 1 and 2 show that 1) few genes are epistatically homogenous, and 2) that but for a subset of highly central nodes corresponding to genes with a unique transcript, alternatively-spliced genes are likely to be more epistatically versatile than genes with a single transcript. Importantly, the distributions of clustering coefficients demonstrate the independence of the genetic interactions; since most genes are not in coherent clusters, knowing their interactions does not inform us about the interactions of their interactors. Thus, it is not possible to predict undetected genetic interactions based on knowledge of existing interactions.

Sequential removal of nodes from the *D. melanogaster* genetic interaction network confirms the different location of the two classes of nodes (Figure 3B). Highly connected alternatively spliced nodes are likely to be hubs, whereas the highly connected nodes with an unique transcript would contain a mixture of hubs and bottlenecks bridging functional groups. The *C. elegans* network has fewer edges and the observed shape of the curves probably arises from the mixed effect of deleting hubs and peripheral nodes. Moreover, the results suggest that there are no strict bottlenecks linking different parts of the network. In biological context, this means that most biological processes are interconnected and any mutation can put several of them under pressure. Interestingly, there is a large difference in the diameter of the networks for the two species, despite the networks having a similar number of nodes. Thus the *C. elegans* network must be more interconnected than that of *D. melanogaster* despite having fewer edges. The gene lin-35 contributes substantially to this effect since it has an unusual high number of genetic interactions. If this gene were a bottleneck, the network would fall apart after the gene’s deletion, greatly diminishing the diameter of the network. However, as no such event is observed, this means that other genes must be highly connected in order to avoid the network dispersion. Indeed, the single deletion of lin-35 raises the diameter from 7 up to 8 links. *D. melanogaster* is a more complex organism than *C. elegans*, as it contains more tissues and cell types. It might be possible that the lowest interconnection is due to higher specialisation of genes and to greater compartmentalisation of the biological processes.

It should be noted that the genetic interaction network is not completely characterised for either organism. Previous research showed that a network subset may differ from the whole networks in their features, particularly the “scale-free” nature [36]. Nevertheless, degree distributions, which are the most biologically relevant in our study, are the network measures both quantitatively and qualitatively less affected by sample biases [37]. Genetic interaction experiments are independent of both the feature (AS) and the criterion to classify nodes (presence or absence of multiple transcripts). Although the features for particular nodes are a by-product of the incompleteness of the data, an implicit assumption is that both classes of nodes would be affected to a similar extent. Thus, we believe that the characteristics of our analyses will help to hold our conclusions when analysing more complete networks.

In summary, we have shown that alternatively spliced genes tend to have more genetic interactions than genes with a single transcript. Thus, AS is not only introducing transcriptome diversity, but also increments gene functional versatility, meaning that one gene may be involved in multiple biological processes. There is no difference between genes with or without AS in terms of their functional homogeneity, nor in terms of functional processes. Nevertheless, alternatively spliced genes are not evenly distributed on the genetic interaction network, suggesting that there must exist other factors that influence the chances of a gene for being affected by AS or not; e.g.: the ability for bridging particular functional processes. As a final point, our analyses demonstrate that the AS-related increase in functional versatility is not species-specific; however, aspects such as organismal complexity could affect the location of genes into the network.

## Supporting Information

Figure S1 **Relationship between degree and betweenness of nodes.** Left, *C. elegans*. Right, *D. melanogaster*. Whole results are in the top row. Low row graphs show a detail of the whole results. In blue, alternatively spliced genes. In red, genes with a unique transcript.
(PDF)
