# Supplementary figures and images for: Analysis of Genetic Interaction Networks Shows That Alternatively Spliced Genes Are Highly Versatile
Source: PLoS One. 2013 Feb 7;8(2):e55671. doi: 10.1371/journal.pone.0055671 (PMC3567133; doi:10.1371/journal.pone.0055671)

*C. elegans*

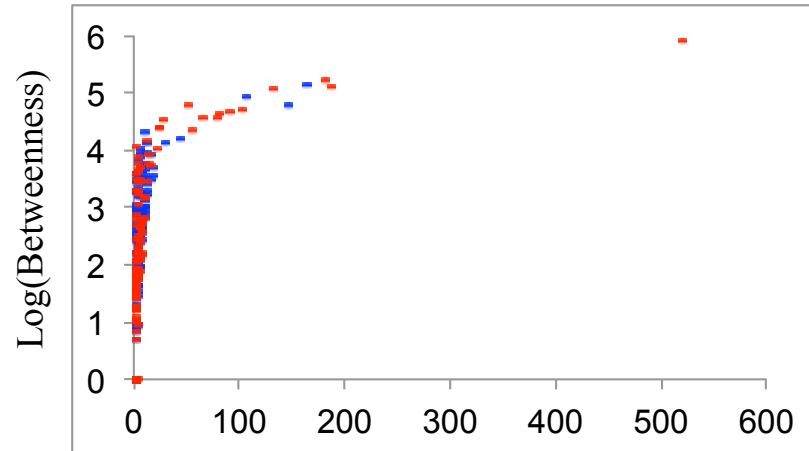

*D. melanogaster*

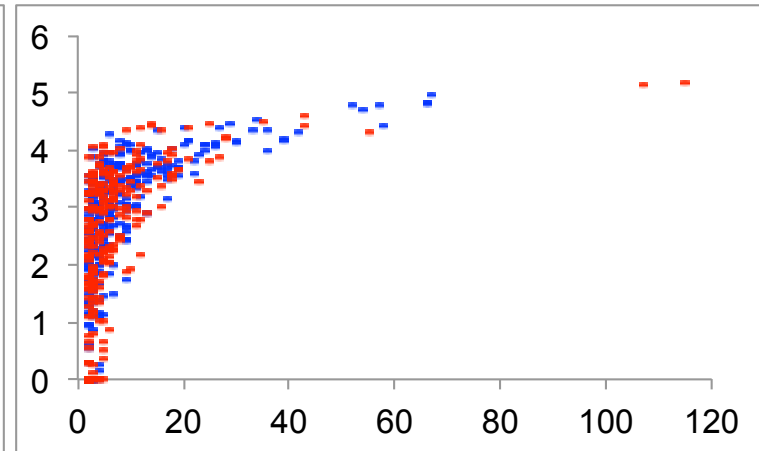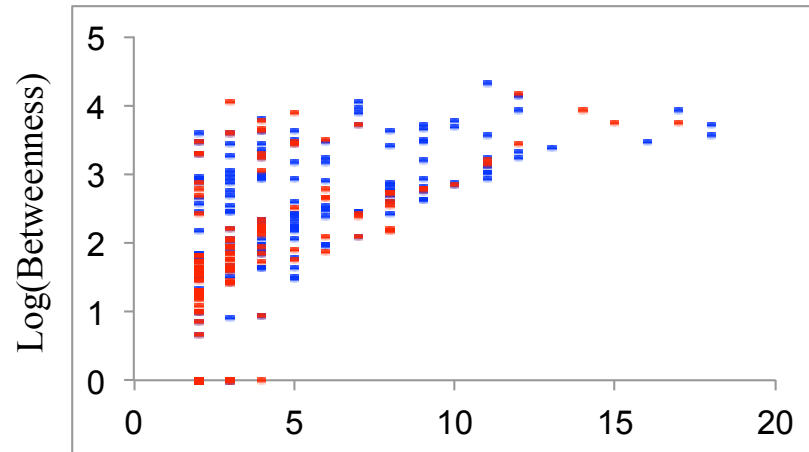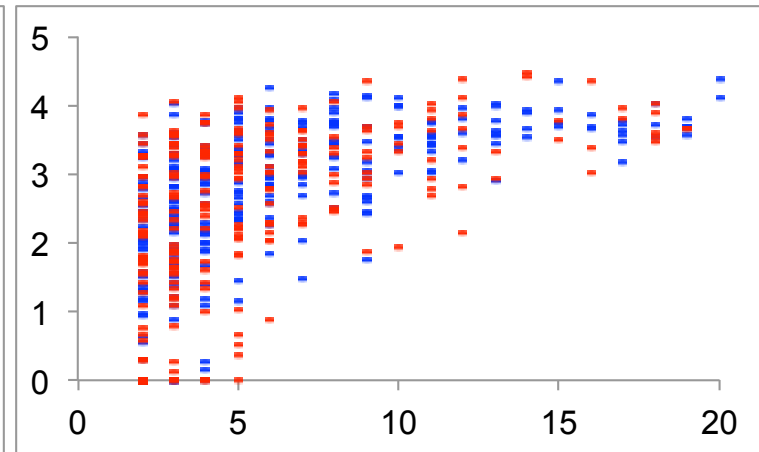

Degree

Degree

Supplement: Figure S1 — Relationship between degree and betweenness of nodes. Left, C. elegans. Right, D. melanogaster. Whole results are in the top row. Low row graphs show a detail of the whole results. In blue, alternatively spliced genes. In red, genes with a unique transcript. (PDF) [file pone.0055671.s001.pdf]
